# Insomnia and excessive daytime sleepiness in medical students: consequences of the use of technologies?

**DOI:** 10.5935/1984-0063.20220008

**Published:** 2022

**Authors:** Gabriela Miloch da Silva Cardoso, Mariana Pires Ferreira Novaes da Silva, Camila de Castro Corrêa, Silke Anna Theresa Weber

**Affiliations:** 1 Faculdade de Medicina de Botucatu, Departamento de Otorrinolaringologia - Botucatu - SP - Brazil.; 2 Centro Universitário Planalto do Distrito Federal, Fonoaudiologia - Brasília - Distrito Federal - Brazil.

**Keywords:** Sleep, Students, Medical, Technology

## Abstract

**Objective:**

To analyze the relation among insomnia, excessive daytime sleepiness and the excessive use of technologies in medical students.

**Methods:**

The study was approved by the Local Ethics Committee of the institution. Students from the 1st and 2nd year of medical graduation students participated. Three questionnaires were used: Sleep Time-Related Information and Communication Technology, Insomnia Severity Index and the Epworth Sleepiness Scale. The data were described and compared by gender and year of graduation by the Students T Test, and correlated to the use of technology, insomnia and excessive daytime sleepiness by Pearsons Correlation (adopted the significance level of *p* <0.05).

**Results:**

106 students (41 male) participated, expressing perception of insomnia in 76.4%, 34% with excessive daytime sleepiness, and 38.3% had a high use of technology related to sleep. There was a correlation between the use of technologies both with insomnia (r = 0.393; *p* = < 0.001), as well as with excessive daytime sleepiness (r = 0.228; *p* = 0.019).

**Conclusion:**

An important frequency of insomnia was found associated with the excessive use of technologies at the bed, with repercussions of daytime sleepiness. This demonstrates the importance of actions to raise awareness and education about correct sleep hygiene in medical students.

## INTRODUCTION

Insomnia is defined as a disorder characterized by the inability to fall asleep, maintain or regain sleep, being prevalent in about 10 to 15% of the general population. The prevalence is higher in older ages, in females, in whites and in people with physical or mental illness^1^.

Meanwhile, excessive daytime sleepiness is characterized by an inability to stay awake and alert during the main periods of wakefulness, interrupted in drowsiness and unintended sleep lapses^2^. It is a complex symptom, more frequent in adolescence and early stage adult (especially university students), due to biological, environmental and behavioral factors. The changes in sleep patterns of university students are associated with increased school/academic obligations and social activities^3^, in addition to the use of technologies and television, collaborating with later bedtimes and more somnolence during the day^4^.

Medical students often experience insomnia, related to the demands of the curriculum. Specifically for students who are in clinical stages, insomnia was observed in 33%, in addition to excessive daytime sleepiness (40%) and poor sleep quality in 30%^5^, reaching up to 61.7%^6^. It was also highlight that insomnia directly influenced academic performance^5^.

With the globalization of technologies, there was easier access to more people, implying a significant increase of technology use in the daily lives of young people, directly correlating with sleep quality in this age group, for example decreasing the sleep duration, increasing the latency of falling asleep and even reaching REM sleep stage^7^. He also found a strong relationship between the use of technology and sleep patterns. The more types of devices were used, the more individuals had difficulty falling asleep and/or maintaining sleep^8^.

About 22% of respondents reported going to sleep with their cell phones on, and 10% reported being woken by their mobile devices at night at least a few times a week (which made it difficult to resume sleep). Different levels of interactivity of the various devices can influence sleep patterns, with more interactive devices suggesting to be more impactful^8^.

Therefore, the importance of analyzing the consequences of the use of technologies on the sleep habits of university students is emphasized, since it is a group with rich access and constant use of technologies, with supposedly poor quality of sleep.

It is expected to observe in medical students that the use of technologies is an aggravating factor for symptoms related to sleep, such as insomnia and excessive daytime sleepiness. In consequence, the present research aimed to analyze the degree of insomnia, quantify the use of technology in university students and investigate the relationship between insomnia and daytime sleepiness with the excessive use of electronics.

## MATERIAL AND METHODS

The study was approved by the Human Research Ethics Committee of the institution involved.

In order to participate in this study, students from the 1st and 2nd year of graduation in medicine at a public university in the interior of São Paulo (180 students in total) were invited. Students who did not want to participate in the study or who did not complete the questionnaires in full were excluded.

The first questionnaire applied was the Sleep Time-Related Information and Communication Technology (STRICT) with 11 questions self-administered, separated in two parts. The first (with 7 questions) quantifying the total use of STRICT (cell phones/text messages, Facebook/Twitter/Instagram, online games, hours of use in bed) and the second (with 4 questions), the sleep duration and bedtime on weekdays and weekends. The questions contain four to six answer options, with higher numbers indicating a greater amount of technology use, and at the end all questions are added (maximum score 43). In addition, a difference was made between the score obtained for sleep duration (question 9 - question 8) and bedtime (question 11 - question 10) at weekends and on weekdays. A positive difference indicates a longer sleep duration and later bedtime on weekends^9^.

The Insomnia Severity Index (IGI) measures self-perception of insomnia, through subjective symptoms and consequences of insomnia, as well as the degree of concern or anguish caused by these factors. Its content corresponds to the insomnia diagnostic criteria, composed of seven items classified on a scale from 0 to 4, and the total score ranges from 0 to 28. The cutoff points were adopted to classify the severity of insomnia: absence of significant insomnia (0-7), lower limit for insomnia (8-14), moderate clinical insomnia (15-21) and severe clinical insomnia (22-28)^10^.

The Epworth Sleepiness Scale (ESE) measures excessive daytime sleepiness by the probability of falling asleep in eight situations of daily activities, some of them highly soporific. The global score ranges from 0 to 24, with scores above 9 indicating excessive daytime sleepiness^11^.

The results of the questionnaires were tabulated and described in percentage, average and standard deviation. All variables were subjected to the Shapiro–Wilk normality test. Inferential statistical analysis was performed, using parametric data to compare sex and year of graduation, through the Student’s T Test. For the correlation between use of technology, perception of insomnia and daytime sleepiness, the Pearson’s Correlation was used. The level of significance was set at p<0.05. The program used was Jamovi, version 1.2.25.

## RESULTS

The study had 106 participants, 41 of them were male, with an average age of 20 ± 1.67 years.

The results obtained at the ESE showed that 36 students (34%) have excessive daytime sleepiness, 24 (67%) of the second year and 27 (75%) women. Regarding the IGI, 81 (76.4%) had perceived insomnia, with 60 students (74%) being classified as mild insomnia, 19 (23,45%) as moderate insomnia, 02 (2,45%) as severe insomnia. When comparing the population by sex and year of graduation regarding ESE and IGI, there was no statistical difference. About the results of technology use there was no statistical difference too (Table 1).

**Table 1.** Mean, standart deviation and association of insomnia, excessive daytime sleepiness (Epworth) and results of technology use, by year of graduation and by sex.

	All (n=106)	Women (n=65)	Men (n=41)	p	1^st^ year (n=51)	2^nd^ year (n=55)	p
**Insomnia**	10.9±4.47	10.4±4.02	11.2±4.74	0.36	11.0±4.61	10.8±4.39	0.81
**Epworth**	8.12±3.78	7.51±3.50	8.51±3.92	0.19	7.51±3.58	8.69±3.90	0.11
**STRICT**	11.1±3.88	11.2±4.08	11.1±3.79	0.95	10.7±3.82	11.5±3.94	0.31
**Nº hours on technology before bedtime**	2.62±1.31	2.61±1.45	2.63±1.22	0.94	2.49±1.25	2.75±1.35	0.32
**Nº hours on technology in bed**	1.85±1.04	1.88±1.12	1.83±0.99	0.82	1.69±0.97	2.00±1.09	0.12

Legend: W - women, M - Men; Student's t-test, (*) significance level p<0.05.

In the STRICT questionnaire, the average sleep duration of the 1st year of graduation was 6h6min during the week, and 7h56min on weekends, while for the 2nd year the average was 6h17min during the week and 8h34min on weekends, however there was no difference (p=0.381). There was also no difference for the bedtimes during the week (p=0.384) and at weekends (p=0.409), as shown in Graph 1.

In the STRICT questionnaire (Sleep Time-Related Information and Communication Technology), it was observed that 37 people (38.3%) had high scores, indicating a greater amount of daily use of technology related to the sleep routine. There was a correlation between the use of technologies with insomnia (r = 0.393; p=<0.001), as well as with ESE (r = 0.228; p = 0.019). The hours of the use of technologies in bed also correlated to ESE (r=0.22; p=0.023) and to insomnia (r=0.24; p=0.014), as shown in Table 2.

**Table 2.** Correlation between insomnia, excessive daytime sleepiness and excessive use of technologies.

Variables	M±SD	1	2	3	4	5
1. Epworth	10.9±4.47					
2. Insomnia	8.12±3.78	0.18				
3. STRICT	11.1±3.88	* 0.23	* 0.39			
4. Nº hours on technology before bedtime	2.62±1.31	-0.02	* 0.26	* 0.59		
5. Nº hours on technology in bed	1.85±1.04	* 0.22	* 0.24	* 0.75	* 0.002	

Legend: M: mean; SD: standart deviation; Pearson's correlation, (*) significance level p<0.05.

## DISCUSSION

The topic of investigating habits related to sleep and the use of technologies is currently relevant due to the exponential increase in behaviors related to this use in the routine of everyone in general. Regarding the population of medical students, this discussion is important due to the high demand during the undergraduate course, in addition to associating these demands with the personal life, in which information and communication technologies are intensely inserted.

The sleep duration of both years of graduation during weekdays was about 1 hour shorter than that reported by young adults in the American population in general, probably due to the intense workload of studies. On weekends it was similar in the 1st year and about 30 minutes higher in the 2nd year. This increase in duration at the weekend can be explained by the compensation of sleep hours in this period. The sleep hours on weekdays were equivalent to those reported in the literature, while the sleeps hours on weekends were longer in half an hour^12^.

Regarding the insomnia parameter, it was observed that the students had an average of intense difficulty falling asleep (34%), staying asleep (23.6%) and waking up earlier than expected (34.9%). These data were like those reported in previous studies with medical students, in which insomnia was expressed in 18.3%^13^, 26%^14^ even in 40%^5^.

It was also found in the IGI questionnaire that 97.1% of the students considered that their sleep problem interfered in their daily functioning in some way, and 46.2% considered this interference as “very” or “intense”. This is similar to what was reported in the previous literature, in which 94% considered interference from lack of sleep in their daily functioning, with 51% considering it as a major impact^12^.

As regards to excessive daytime sleepiness, there was no progression between the 1st and 2nd year. The expressive percentage of excessive daytime sleepiness observed in freshmen (34%), may reflect the adapting process to graduation. In addition, students had an exhaustive study schedule or intensive preparatory courses for the entrance exam, which could indicate a previous maladjusted pattern^15^. In studies with pre-university students, an even greater occurrence of excessive daytime sleepiness, of 45,9%15 and 55,8%^16^, was observed.

All university students reported using technology before bedtime, varying the time of use, but 41.5% of students used an electronic device for at least 1 hour before sleep. The excessive use of technology, and the hours of technologies in bed, were significantly associated with excessive daytime sleepiness and insomnia, which can negatively affect individuals in several academic areas, as previously reported^5,9,13,14^.

It is noteworthy that the present study had limitations in terms of not having an objective sleep test, the polysomnography, to compare electrophysiological parameters with the behavior of sleep hygiene related to the use of technologies. In addition, the sample was restricted to only one undergraduate course in the health area and two years of this course, although, it was possible to observe the presence of important changes in sleep patterns and factors that may interfere with sleep.

It should be noted that studies enrolling the medical students are important and should be intensified, comparing the subjective sleep parameters to other courses, verifying that the losses in the medical population are more intense^17^. Two previous studies report that, in general, medical students showed changes in their sleep quality, however the initial phases of the course expressed the most important ones, even though the subsequent phases have a greater academic demand^3,18^. This inversion points out the possible change in the perception of subjective sleep parameters during the curriculum/medical career.

Thus, it is recommended to carry out systematized actions of health promotion to raise awareness of behaviors which favor a good sleep hygiene in this population^19,20,21,22^.

The study found a high frequency of insomnia, associated with the excessive use technologies, in general and in bed, with repercussion of excessive daytime sleepiness. Early intervention of promotion of sleep hygiene and improving the awareness with the use of technologies is highly recommended in medical students.
